# Integrative Genome-Wide Gene Expression Profiling of Clear Cell Renal Cell Carcinoma in Czech Republic and in the United States

**DOI:** 10.1371/journal.pone.0057886

**Published:** 2013-03-05

**Authors:** Magdalena B. Wozniak, Florence Le Calvez-Kelm, Behnoush Abedi-Ardekani, Graham Byrnes, Geoffroy Durand, Christine Carreira, Jocelyne Michelon, Vladimir Janout, Ivana Holcatova, Lenka Foretova, Antonin Brisuda, Fabienne Lesueur, James McKay, Paul Brennan, Ghislaine Scelo

**Affiliations:** 1 International Agency for Research on Cancer (IARC), Lyon, France; 2 Palacky University, Olomouc, Czech Republic; 3 Charles University in Prague, First Faculty of Medicine, Institute of Hygiene and Epidemiology, Prague, Czech Republic; 4 Department of Cancer Epidemiology and Genetics, Masaryk Memorial Cancer Institute, Brno, Czech Republic; 5 Department of Urology, Second Medical Faculty, University Hospital Motol, Prague, Czech Republic; Johns Hopkins University, United States of America

## Abstract

Gene expression microarray and next generation sequencing efforts on conventional, clear cell renal cell carcinoma (ccRCC) have been mostly performed in North American and Western European populations, while the highest incidence rates are found in Central/Eastern Europe. We conducted whole-genome expression profiling on 101 pairs of ccRCC tumours and adjacent non-tumour renal tissue from Czech patients recruited within the “K2 Study”, using the Illumina HumanHT-12 v4 Expression BeadChips to explore the molecular variations underlying the biological and clinical heterogeneity of this cancer. Differential expression analysis identified 1650 significant probes (fold change ≥2 and false discovery rate <0.05) mapping to 630 up- and 720 down-regulated unique genes. We performed similar statistical analysis on the RNA sequencing data of 65 ccRCC cases from the Cancer Genome Atlas (TCGA) project and identified 60% (402) of the downregulated and 74% (469) of the upregulated genes found in the K2 series. The biological characterization of the significantly deregulated genes demonstrated involvement of downregulated genes in metabolic and catabolic processes, excretion, oxidation reduction, ion transport and response to chemical stimulus, while simultaneously upregulated genes were associated with immune and inflammatory responses, response to hypoxia, stress, wounding, vasculature development and cell activation. Furthermore, genome-wide DNA methylation analysis of 317 TCGA ccRCC/adjacent non-tumour renal tissue pairs indicated that deregulation of approximately 7% of genes could be explained by epigenetic changes. Finally, survival analysis conducted on 89 K2 and 464 TCGA cases identified 8 genes associated with differential prognostic outcomes. In conclusion, a large proportion of ccRCC molecular characteristics were common to the two populations and several may have clinical implications when validated further through large clinical cohorts.

## Introduction

Renal cell carcinoma (RCC) is a constellation of malignancies of different histological subtypes arising from the renal parenchyma. The most common histological subtype of RCC is clear cell RCC (ccRCC) which accounts for 70–80% of sporadic cases and for the majority of renal cancer mortality [1]. Worldwide in 2008 more than 273,000 cases of renal cancer have been diagnosed and 116,000 patients died of this cancer. Several countries in Central and Eastern Europe show high rates of renal cancer incidence and mortality. The Czech Republic has reported the highest incidence and mortality rates in the world with age-standardized incidence and mortality rates of 16.6 and 5.6 per 100,000 person-years, respectively [2].

Several risk factors associated with ccRCC have been identified in large epidemiological studies, such as cigarette smoking, obesity, hypertension (and antihypertensive medication) and family history of the disease [3]. On the molecular level, RCC is a heterogeneous disease controlled by different genes and pathways. Recent developments driven by advances in genomic biology have brought a significant body of knowledge into our understanding of this disease. Loss-of-function alterations in the von Hippel Lindau (*VHL*) tumour suppressor gene have been observed in at least two-thirds of sporadic ccRCC tumour tissue [4], and germline mutations involved in hereditary ccRCC as well [5]. Inactivating mutations of other tumour suppressor genes, such as *SETD2*, *KDM6A*, *KDM5C*, *PBRM1* and *BAP1* have recently been reported to play a role in ccRCC carcinogenesis. For all but *BAP1*, the proteins encoded by these genes are involved in histone and chromatin regulation [6], [7], [8]. Hypoxia-inducible factors (HIF) pathway and compensatory hyperactivation of angiogenesis through upregulation of VEGFR and PDGFR pathways are thought to be particularly important in ccRCC pathogenesis, given the highly vascularised nature of renal tumours and the specific association with mutations in *VHL* [9]. In addition, activation of PI3K/AKT/mTOR [10], Wnt/β-catenin [4], [11], epithelial to mesenchymal transition (EMT) [12], [13], HGF/MET [11], [14] as well as inflammatory [15] pathways have all been reported to be implicated in RCC carcinogenesis.

Transcriptional profiling has emerged as a powerful approach for identification of molecular subgroups (class discovery), prediction and relation to clinical outcome in many cancers. In ccRCC several studies identified two patterns of gene expression with different cancer-specific survival [16], [17], [18]. Others have reported transcript patterns related to expression of HIF1 and HIF2 factors, regulated by VHL [6], [19] and gene signature associated with metastatic potential [20], [21]. To our knowledge, populations from the Central and Eastern Europe have not been covered in previous reports, which mostly included limited number of cases and internal replications. Using the array-based whole genome gene expression technology, we performed a gene expression profiling of 101 ccRCC specimens and their adjacent non-tumour renal tissue collected in patients from the Czech Republic to explore systematically the molecular variations underlying the biological and clinical heterogeneity of this cancer. In parallel, we performed secondary statistical analysis of RNA sequencing data generated by The Cancer Genome Atlas (TCGA) consortium in an effort to replicate our findings in an independent ccRCC patient series from the US.

## Results

### Unsupervised Hierarchical Clustering (K2 Series)

**Figure 1:**
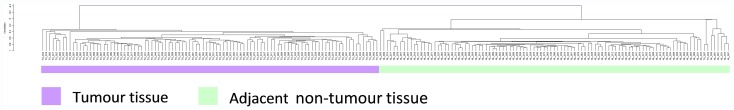
Unsupervised Hierarchical Clustering of K2 samples. Unsupervised Hierarchical Clustering of all samples following quantile normalization highlighting two groups of samples: tumour (violet) and adjacent non-tumour (light green) tissue. Dendrogram for clustering experiments was created using centred correlation and average linkage method. Length of nodes corresponds to correlation between samples.

To compare the gene expression profiles of all 101 tumour and adjacent non-tumour tissue sample pairs of the K2 series (described in Table 1), we first performed an unsupervised hierarchical cluster analysis of vst-transformed and quantile normalized gene expression data without background subtraction using all 47,231 probes present in the dataset (corresponding to 28,688 well-established coding transcripts). In unsupervised clustering of tumour and non-tumour tissue, all tumour samples clustered together: the dominant distinction was between tumour and non-tumour tissues rather than between individuals (Figure 1). Furthermore, we examined the expression profiles of tumour and adjacent non-tumour tissue samples separately (Figure S1). We found that all tumour samples were tightly clustered together suggesting homogeneity of ccRCC samples used in this study. Similarly, we did not observe significant differences between adjacent non-tumour tissue samples. There was also little evidence of any batch effects (day the arrays were performed, lot number of chip), or difference by RNA quality levels, percentage of viable tumour cells and processing procedures at local recruiting centres that may be confounding the results (data not shown).

**Table 1:** Characteristics of the 101 ccRCC patients from the “K2 study” (Czech Republic) included in the whole-genome gene expression microarray study.

Characteristics	Male			Female		p-value*	
	N	%	N		%		
**Total (N = 101)**	59	100	42		100		
**Recruiting Center**							
**Czech Republic**							0.685
Ceske Budejovice	28	47.5	22		52.4		
Prague	14	23.7	6		14.3		
Brno	10	17.0	9		21.4		
Olomouc	7	11.9	5		11.9		
**Age**							**0.060
42–44							
45–54	15	25.4	5		11.9		
55–64	22	37.3	11		26.2		
65–74	13	22.0	19		45.2		
75–84	9	15.3	7		16.7		
**Age,** Mean ± SD	62.8 (±10.0)		65.8 (±7.9)				0.108
**Body mass index (BMI) 2 yrs prior to recruitment**							0.985
22.0–24.9	12	20.3	8		19.1		
25–29.9	26	44.1	20		47.6		
30–47.3	21	35.6	14		33.3		
**Body mass index (BMI)** [kg/m2], Mean ± SD	28.95 (±4.7)		28.44 (±3.9)				0.567
**Grade (Fuhrman system)**							0.919
Well-differentiated	14	23.7	8		19.1		
Moderately differentiated	27	45.8	20		47.6		
Poorly differentiated	13	22.0	11		26.2		
Undifferentiated	5	8.5	3		7.1		
**Stage** ***							0.190
I	28	47.5	15		35.7		
II	6	10.2	8		19.0		
III	7	11.9	4		9.5		
IV	5	8.5	9		21.4		
Missing	13	22.0	6		14.3		
**Smoking status**							0.065
Never	24	40.7	27		64.3		
Former (>1 yr since quitting)	12	20.3	5		11.9		
Current	23	39.0	10		23.8		
**Self-reported hypertension history**							0.713
Yes	40	67.8	27		64.3		
No	19	32.2	15		35.7		
**Treatment**							
**First line treatment**							0.120
Radical nephrectomy	55	93.2	35		83.3		
Partial nephrectomy	4	6.8	7		16.7		
**Second line treatment**							
None	51	86.4	32		76.2		
Antiangiogenic and/or biotherapy	6	10.2	6		14.3		
Radiotherapy and/or chemotherapy	1	1.7	1		2.4		
Additional surgery	1	1.7	1		2.4		
Combination of the above	0	0.0	2		4.8		

* p value calculated using Pearson χ^2^ testing for categorical variables and t-test for continuous variables.

** The two younger categories were grouped.

*** All stage IV patients had distant metastasis at diagnosis, and by definition none of stage I, II or III patients had distant metastasis. Missing stages were due to the lack of lymph nodes and/or metastasis evaluation. Out of 19 cases with missing stage, 9 were pT1a, 7 were pT1b, 1 was pT2a, and 1 was pT3a.

### Identification of Differentially Expressed Genes and Pathway Analyses (K2 Series)

**Figure 2:**
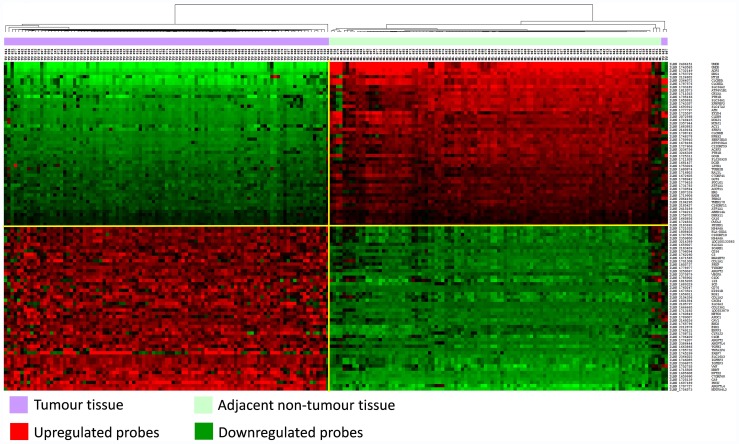
Hierarchical Classification of 100 significant differentially expressed genes in K2 series. Heatmap representing the 50 significant upregulated (in red) and 50 downregulated (in green) probes with the highest fold change following differential expression in ccRCC compared with non-tumour tissue (FDR-adjusted p-value (BH) <0.05, FC ≥2).

We conducted paired analysis on the samples from the 101 K2 patients to identify genes differentially expressed in tumour vs. adjacent non-tumour tissue using the genome-wide expression microarray data. This comparison resulted in 1650 significant differentially expressed probes (modulated by at least 2-fold [FC ≥2] and showing an Benjamini and Hochberg (BH) false discovery rate (FDR) adjusted p-value <0.05 for the paired t-test comparison, Table S1), of which 743 probes were upregulated and 907 were downregulated in tumour samples, mapping to 630 and 720 unique genes, respectively. Hierarchical clustering analysis restricted to 50 the most up-, and 50 the most down - regulated probes correctly classified tumour and non-tumour samples in two groups, with the exception of two tumour samples (Figure 2). The biological characterization of the 1350 differentially expressed genes using Gene Ontology (GO) annotation implemented in the DAVID programme (http://david.abcc.ncifcrf.gov/) highlighted that the majority of genes downregulated in ccRCC tissues were involved in metabolic processes (organic, carboxylic, amino acid, lipid), catabolic processes, oxidative reduction, excretion, ion transport, response to chemical stimulus and cellular localization. The genes upregulated in ccRCC tissues were associated with regulation of immune and inflammatory responses, response to stimuli, stress, wounding and hypoxia, regulation of cell proliferation, cell activation, angiogenesis, cell adhesion and motility. The complete list of enriched GO terms and genes is available in Table S2.

To gain further insight into the biological pathways involved in ccRCC pathogenesis, a gene set enrichment analysis (GSEA) was performed. GSEA (http://www.broadinstitute.org/gsea/) is a powerful bioinformatics tool that determines whether *a priori* defined sets of genes (“pathways”) show statistically significant, concordant differences between two biological states (e.g. phenotype). We found 84 pathways to be enriched in ccRCC phenotype at FDR cut-off values of 0.25 [22] using the Biocarta pathway database from the Molecular Signatures Database – MsigDB (http://www.broad.mit.edu/gsea/msigdb/index.jsp). These included cell cycle pathways (G1, G2, CELLCYCLE) characterized by the presence of cyclins *(CCND1, CCND2, CCND4, CCNB1*), cyclin-dependent kinases (*CDK2, CDK4, CDK6, CDK7*), cyclin dependent kinase inhibitors (*CDKN1A, CDKN1B, CDKN2A, CDKN2B, CDKN2C, CDKN2D*) as well as retinoblastoma 1 (*RB1*), *CHEK1* and *CHEK2*. Furthermore, GSEA identified several oncogenic signalling pathways, such as ARF, P53, ATM, MET, ATRBRCA, WNT and ETS pathways. The top-score genes recurring in these pathways included key cancer genes, such as *MYC, RB1, TP53, JUN, ATM, CDKN1A, NFKB1* among others. Other pathways known to be associated with ccRCC have been identified including gene sets linked to hypoxia (HIF pathway), angiogenesis (VEGF pathway) as well as immune and inflammatory signalling (INFLAM, TOLL, TH1TH2, NFKB, NKCELLS, TCR, DC, TNFR1, TNFR2, CYTOKINE) pathways. The genes related to inflammation and immune response enriching the mentioned pathways included cytokines and their receptors (*IL2, IL4, IL6, IL8, IL10, IL12, IFNG*), toll-like receptors (*TLR2, TLR3, TLR4, TLR6, TLR7, TLR10*), NF-κB genes (*RELA, NFKB1, NFBIA, TNF, TNFAIP3*) as well as T-cell signalling genes (*ZAP70, LCK, LAT, FYN CD3D, CD3E, CD3G*). In addition, gene sets related to survival, apoptosis (FAS, PML, DEATH, CASPASE and BAD) and kinase signalling (GSK3, P38MAPK, PAR1, PTEN, ERK) were enriched in ccRCC phenotype. The complete list of pathways is included in Table S3.

### Replication of Differential Expression in an Independent TCGA Population

To explore whether the ccRCC gene expression signatures identified in the Czech Republic population were reproducible in other populations and using a different experimental platform, we evaluated the gene expression status of 65 tumour/non-tumour renal tissue pairs from ccRCC patients recruited in the US for the TCGA project and sequenced through the RNA sequencing (RNA-seq) technology. Key clinical characteristics of the cases available are described in Table 2. The Level 3 processed RNA-Seq TCGA data, generated by an Illumina HiSeq2000 platform, were downloaded from https://tcga-data.nci.nih.gov/tcga/. The flowchart of the previous analysis performed by the TCGA data analysis centres and further data normalization and transformation are presented in Figure S2A and S2B, respectively. We used RPKM values [23] calculated by (raw read counts×10^9^)/(total reads×length of a gene) as an estimate of gene expression. The RPKM method corrects for biases in total gene exon size and normalizes for the total short read sequences obtained in each tissue library. RPKM values for each sample were merged into a single file, TMM normalized (similar assumptions to quantile normalization) [24] and log_2_-transformed. Following the recommendations provided in edgeR and limma packages on the use of raw count data in the mentioned methods, the same analysis was performed using both raw count as well as RPKM values. Both analyses resulted in the comparable set of most differentially expressed genes (BH FDR-adjusted p-values <0.05 and FC ≥2); however, more significant differentially expressed genes were obtained when using count values. Taking into consideration that significant genes found using the RPKM method were overlapping with count data analysis, this set of genes was used for all downstream analysis. As with the microarray analysis described above, genes with BH FDR-adjusted p-values <0.05 and FC ≥2 were considered. Using paired t-tests for the complete set of genes (20,532), we identified 2493 significant differentially expressed genes (Table S4), of which 1299 genes were upregulated and 1194 genes were downregulated. To obtain a broader overview of the biological changes occurring at the gene expression level, we used Gene Ontology term enrichment (DAVID software) to identify annotated functions and processes that were overrepresented in genes that were significantly up -or down -regulated. Likewise in microarray analysis, downregulated genes were involved in metabolic and catabolic processes, excretion, ion transport, oxidation-reduction, localization and developmental processes (including kidney development). Besides, the upregulated genes enriched mainly immune system responses and regulation, inflammation, response to stimulus, cell activation (primarily T-cell activation), antigen processing and presentation, lymphocyte differentiation, angiogenesis and signal transduction (Table S5). Using GSEA on the TCGA RNA-Seq dataset we did not find significant overrepresented pathways (data not shown).

**Table 2:** Characteristics of the 65 ccRCC patients included in the TCGA series (US) using RNA-Sequencing technology.

Characteristics	Male			Female			p-value*
	N	%	N		%		
**Total (N = 65)**	46	70.8	19		29.2		
**Age**						**0.375	
38–44	4	8.7	2		10.5		
45–54	9	19.6	3		15.8		
55–64	14	30.4	4		21.1		
65–74	10	21.7	8		42.1		
75–90	9	19.6	2		10.5		
**Age**, Mean ± SD	62.87 (±12.9)		62.84 (±11.2)			0.994	
**Grade**						***0.358	
Well-differentiated	0	0	1		5.3		
Moderately differentiated	16	34.8	8		42.1		
Poorly differentiated	20	43.5	6		31.6		
Undifferentiated	9	19.6	3		15.8		
Missing	1	2.2	1		5.3		
**Stage** □						***0.821	
I	16	34.8	7		36.8		
II	6	13.0	1		5.3		
III	11	23.9	5		26.3		
IV	10	21.7	5		26.3		
Missing	3	6.5	1		5.3		

Data from all paired tumour/non-tumour sets available on April 19, 2012 were retrieved from TCGA data portal.

* p value calculated using Pearson χ^2^ testing for categorical variables and t-test for continuous variables.

** the younger two categories were grouped.

*** excluding missing category.

□ Of stage IV patients 15 had distant metastasis at diagnosis, and by definition none of stage I, II or III patients had distant metastasis. Missing stages were due to the lack of lymph nodes and/or metastasis evaluation>. These four patients were pT1aNXMX, pT3aNXM0, pT1bNXMX and pT3aNXMX, respectively.

**Figure 3:**
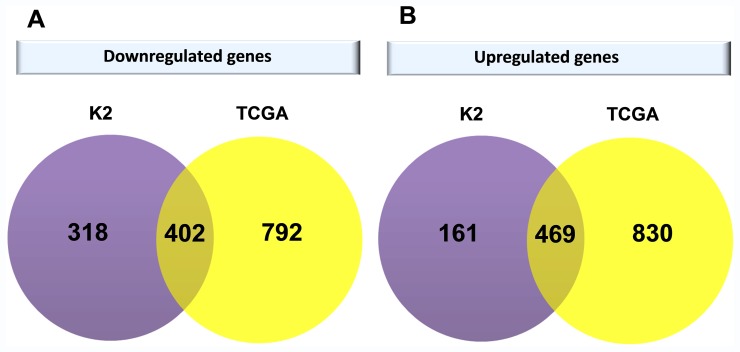
Venn diagrams showing the intersection of significant genes differentially downregulated (A) and upregulated (B) in the whole – genome expression profiling microarray dataset (K2) vs. RNA-Sequencing dataset from the Cancer Genome Atlas (TCGA).

We then investigated the correlation between the RNA-Seq data and the microarray K2 study. A Venn diagram was used to represent the overlap between significant differentially expressed genes obtained with both platforms (Figure 3). RNA-Seq results broadly agree with gene expression measurements obtained with previous microarray data, with approximately 60% (402) of downregulated and 74% (469) of upregulated genes from the discovery phase having been validated in the RNA-Seq analysis. Importantly, in the majority of cases the subset of genes with the highest fold change identified through both analyses overlaps, highlighting the ability of both approaches to accurately record changes in gene expression arising in ccRCC. The 50 genes identified by both methods with the highest (logFC<−3) decrease in expression when compared to adjacent non-tumour tissue included uromodulin (*UMOD*), aquaporin 2 (*AQP2*), kininogen 1 (*KNG1*), chloride channel Ka gene (*CLCNKA*), metallothionein 1G (*MT1G*), ATPases - *ATP6V1B1* and *ATP6V0A4*, ion transport regulator – *FXYD4*, claudin 8 (*CLDN8*), potassium inwardly-rectifying channel gene (*KCNJ1*), T-cell differentiation gene (*MAL*), nephrosis 2 (*SFRP2*), sodium channel gene (*SCNN1A*) and serpin peptidase inhibitor (*SERPINA5*). This indicates that genes important for proper kidney function, responsible for cellular ion homeostasis and transport are deregulated during disease development. The 50 genes overexpressed in the process of ccRCC growth with the highest fold change (logFC >2) included NADH dehydrogenase - *NDUFA4L2*, angiopoietin (*ANGPTL4*), *PNCK* kinase, carbonic anhydrase IX (*CA9*), neuronal pentraxin II (*NTPX2*), nicotinamide N-methyltransferase (*NNMT*), genes encoding glycoproteins *VWF* and *STC2*, insulin-like growth factor binding protein 3 (*IGFBP3*), solute carrier family genes (*SLC16A3* and *SLC6A3*), fatty acid binding protein 7 (*FABP7*), tumor necrosis factor, alpha-induced protein 6 (*TNFAIP6*) and transforming growth factor (*TGFBI*). In addition, cytochrome P450 gene (*CYP2J2*), pyrophosphatase/phosphodiesterase (*ENPP2*), endothelial cell-specific molecule 1 (*ESM1*), transmembrane collagen (*COL23A1*), chemokine (C-X-C motif) receptor 4 (*CXCR4*), regulator of G-protein signaling 1 (*RGS1*), cytokine *CD70* and *SCD* encoding an enzyme involved in fatty acid biosynthesis had also increased expression in both analysis. Furthermore, genes previously associated with ccRCC susceptibility or carcinogenesis, apolipoprotein C-I (*APOC1*), vascular endothelial growth factor A (*VEGFA*), scavenger receptor class B, member 1 (*SCARB1*) were found to be highly overexpressed in tumour tissue in both analysis.

### DNA Methylation Status in ccRCC (TCGA Series)

Based on the hypothesis that DNA methylation modulates an important proportion of gene expression, we compared DNA methylation patterns in ccRCC and adjacent non-tumour renal tissues from 317 TCGA cases to identify which differentially expressed genes were epigenetically regulated. A total of 188 pairs had been analyzed on the Illumina Infinium Human Methylation 27K platform, and 129 non-overlapping pairs on the 450K platform (File S1). Both series had the same sex distribution and included 65% male and 35% female patients. A significant difference of the age distribution between the 2 series was observed (p = 0.001), with a mean of 57 and 62 years in males, and 64 and 65 years in females for the 27K and 450K series, respectively. In both series approximately 80% of patients were diagnosed with moderately (grade 2) or poorly (grade 3) differentiated tumours.

**Figure 4:**
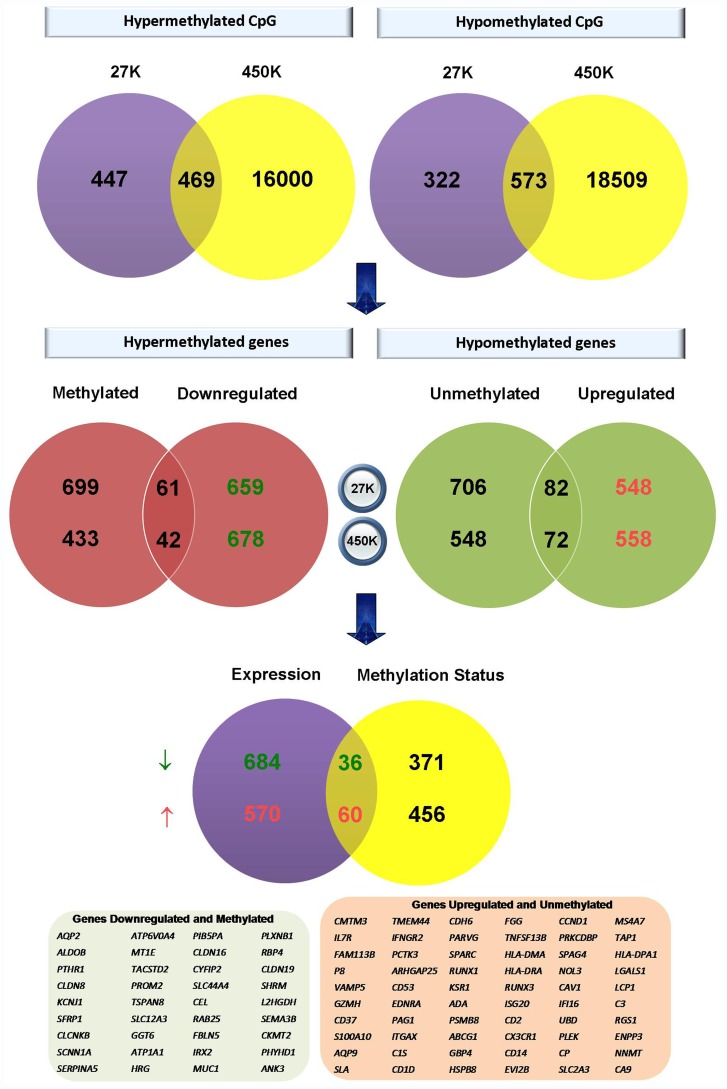
Analysis of ccRCC methylation profile. Flowchart representing analysis of ccRCC methylation profile for Illumina Infinium HumanMethylation 27K (violet) and 450K (yellow) BeadChips showing the intersection of differentially methylated CpG sites (upper panel) and the intersection of significantly (un)methylated genes common to both platforms with differentially expressed genes in the K2 series analysed with Illumina HumanHT-12 v4 Expression BeadChips.

We first compared the DNA methylation patterns in ccRCC versus corresponding non-tumour renal tissue using the following thresholds: FC ≥2 and BH FDR-adjusted p-value <0.05. A total of 916 and 16,469 CpG sites were hypermethylated, and 895 and 19082 were hypomethylated in ccRCC in Illumina Infinium Human Methylation 27K and 450K, respectively (Figure 4, Table S6). Only CpG sites present in both platforms were used for the downstream analysis. We further analyzed the group of significant genes that overlapped with respect to differential DNA methylation (each platform separately) and mRNA expression (microarray). This analysis returned 36 significant genes common to both platforms that were hypermethylated and transcriptionally downregulated (61 and 42 hypermethylated genes for 27K and 450K platforms, respectively) and 60 genes that were hypomethylated and transcriptionally upregulated (82 and 72 hypomethylated genes for 27K and 450K platforms, respectively) (Figure 4). Only 5/96 genes of which expression was epigenetically regulated had not been validated by RNA-Seq analysis. The genes that were downregulated possibly due to hypermethylation events include genes involved in cellular transport (*AQP2, KCNJ1, CLCNKB, SCNN1A, SLC12A3, ATP1A1, CEL, RAB25*), homeostasis (*PTH1R*, *ATP6V0A4*, *CLDN16*), cellular responses (*ALDOB*, *MUC1*, *RBP4*), adhesion (*CLDN8*, *CYFIP2*, *FBLN5*, *CLDN19*), development (*SFRP1*, *PLXNB1*, *SEMA3B*, *ANK3*) as well as cellular and metabolic processes (*SERPINA5*, *MT1E*, *TACSTD2*, *TSPAN8*, *GGT6*, *HRG*, *CEL*, *CKMT2*). The hypomethylated genes included genes that have been previously reported to be associated with ccRCC, such as *CA9*, *NNMT*, *CAV1* and *CCND1*. However, to our knowledge this is the first report on methylation status of *CAV1* in ccRCC. In addition, other upregulated and hypomethylated genes encompass immune response genes (*ENPP3, LCP1, HLA-DPA1, HLA-DRA, HLA-DMA, TNFSF13B, GBP4, PAG1, SLA, AQP9, CD37, IL7R*), genes involved in signal transduction (*RGS1, KSR1, ITGAX, CD53, IFNGR2, S100A10*), cell proliferation *(CCND1, ISG20, SPARC, RUNX3*) and cell death (*LGALS1, PLEK, IFI16, NOL3, CD14, ADA, HSPB8, GZMH*). This data could partly explain the mechanisms leading to up and down regulation of approximately 7% of differentially expressed genes.

### Identification of Gene Signatures for ccRCC Grades (K2 and TCGA Series)

**Figure 5:**
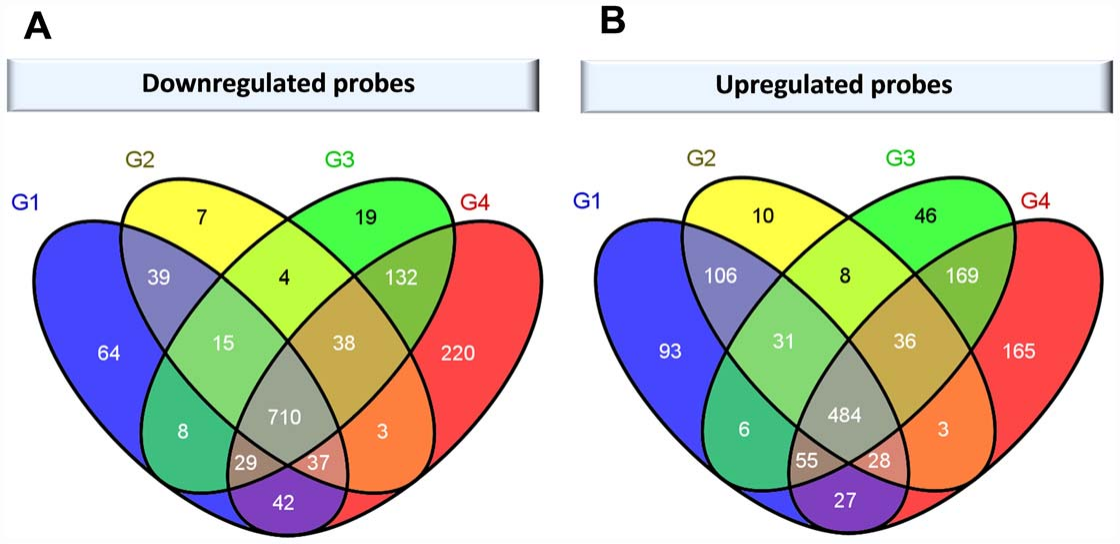
Venn diagrams representing relationship between ccRCC grades and the number of differentially expressed probes. Downregulated (A) and upregulated (B) probes with fold-change (FC) ≥2 and FDR-adjusted p-value (BH) <0.05 using paired sample information are presented.

In many cancers grading is a measure of aggressiveness of the disease and malignancy. The Fuhrman system is used for grading renal cancer into four malignancy level (G1–G4) reflecting levels of differentiation, namely well- (G1), moderately- (G2), poorly- (G3) and un-differentiated (G4). To investigate whether there was a grade specific transcriptional signature in ccRCC we compared the expression levels in cancer tissue and the corresponding adjacent non-tumour tissue for each grade separately based on the microarray data. Out of the 101 K2 cases, 22 were well differentiated (G1), 47 moderately differentiated (G2), 24 poorly differentiated (G3) and 8 undifferentiated (G4). Overall, there were 710 and 484 probes that were commonly down- and upregulated in all the four grades. The differential expression analysis for each grade showed 6.8% (64/944) and 11.2% (93/830) probes uniquely down- and upregulated in G1 grade, 0.8% (7/853) and 1.4% (10/706) in G2, 2% (19/955) and 5.5% (46/835) in G3, and 18.2% (220/1211) and 17.1% (165/967) in G4 (Figure 5, Table s7). These include probes which expression levels show at least 2-fold change and statistical significance of BH FDR-adjusted p-value <0.05. Looking at the number of genes, we observed that there was a general pattern for both down- and upregulated genes, mainly the increased number of solely differentially expressed genes in grades G1 and G4 relative to G2 and G3. This might indicate that the subset of altered genes specific to well differentiated tumours is lost during changes in differentiation. As expected less differentiated cancers which are expected to be more aggressive showed more differentially expressed genes (Figure 5). Similar trends were observed in grade analysis of 65 TCGA cases; however, the majority of grade-specific differentially expressed genes were non-overlapping in both datasets (data not shown). This lack of overlap could be due to distinct grade distribution in both datasets, as well as a poorly reproducible grade assessment of renal tumours [25], [26].

### Gene Expression-based Survival Predictor

Survival data were available for 89 out of 101 ccRCC cases from the K2 series (Czech Republic) and 464 out of 465 TCGA (US) cases. There were significant differences in age and grade distribution between both populations (Table 3). The TCGA series had a longer follow-up duration and higher number of events (deaths) than the K2 series.

**Table 3:** Characteristics of ccRCC patients in the K2 (Czech Republic) and TCGA (US) series included in survival analysis.

Characteristics	K2 Series		TCGA Series				p-value*
	N	%		N	%		
**Total (N = 553)**	89			464			
**Gender**						0.215	
Male	52	58.4		303	65.3		
Female	37	41.6		161	34.7		
**Age**						0.030	
26–44	1	1.1		50	10.8		
45–54	16	18.0		103	22.2		
55–64	30	33.7		138	29.7		
65–74	28	31.5		108	23.3		
75–90	14	15.7		65	14.0		
**Age**, Mean ± SD	64.12 (±9.2)			60.50 (±12.1)	12.13	0.008	
**Grade**						**<0.001	
Well-differentiated	18	20.2		7	1.5		
Moderately differentiated	40	44.9		193	41.6		
Poorly differentiated	23	25.8		172	37.1		
Undifferentiated	8	9.0		67	14.4		
Missing	0	0		25	5.4		
**Extent of primary tumour (pathological assessment)**						0.002	
pT1: ≤7 cm and limited to kidney	55	61.8		228	49.1		
pT2: >7 cm and limited to kidney	18	20.2		57	12.3		
pT3: extends to major veins or perinephric tissues	16	18.0		168	36.2		
pT4: invades beyond Gerota’s fascia	0	0		11	2.37		
**Vital Status**						<0.001	
Alive	77	86.5		312	67.2		
Dead	12	13.5		152	32.8		
**Follow-up duration**, Range (years)	0.5–2.2			0.0–9.2			
**Person-years in follow-up**	112.5			1431			

* p value calculated using Pearson χ^2^ testing for categorical variables and t-test for continuous variables.

** excluding missing category.

**Figure 6:**
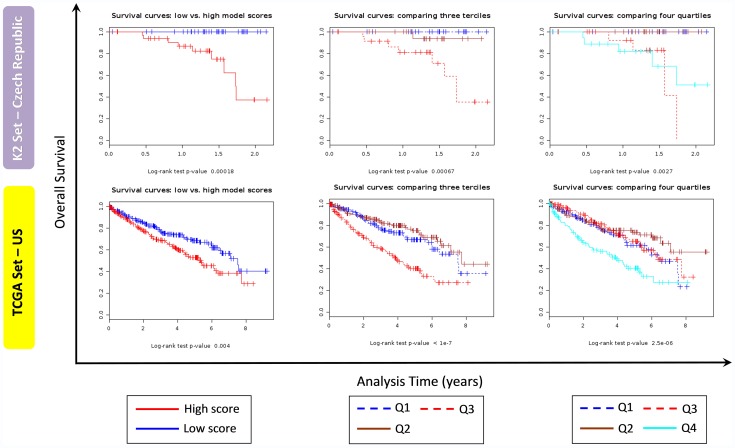
Survival curves for K2 and TCGA series. Survival curves for K2 series (gene expression microarray data, upper panel) and TCGA series (RNA-seq data, bottom panel) using SignS software splitting scores in two groups (first column), three groups (second column) and quartiles (third column). Note: different scale for analysis time axis was used for K2 and TCGA series.

We conducted survival analysis to model overall survival (OS, all-cause mortality) against gene expression levels in tumour tissues in the microarray data from K2 series. Gene expression data of 89 ccRCC cases were examined in an univariate (gene-by-gene) Cox model after FCMS (filter, cluster and stepwise model selection) method using SignS tool [27] to determine the prognostic value of the gene expression status. The obtained model was tested on the 464 RNA-Seq ccRCC TCGA samples. Fifty-one genes correlated positively or negatively with OS (p<0.001) across 10 cross-validated runs. Both the K2 and TCGA series, comparing the survival curves for two, three and four groups suggest that patients fall into two groups (Figure 6). In addition, to evaluate whether important features were missed in the smaller gene expression microarray data, we built a separate model using only the TCGA data, with significance assessed by cross-validation. Two hundred forty six genes correlated positively or negatively with OS (p<0.001) across 10 cross-validated runs in TCGA dataset resulting in 32 genes that were common to both analyses (File S2).

Individual Cox proportional-hazard analysis of selected 51 genes with adjustment for age, tumour grade, extent of primary tumour (pathological assessment: pT) and sex revealed that 8 of these genes were associated with survival independently of age, grade and pT (Table S8). More comprehensive prognosis predictive indexes, such as MSKCC risk score (known as Motzer criteria [28]) for metastatic and UCLA Integrative staging system [29] for localized disease, were not used due to scarcity of available data (in particular ECOG performance status, Karnofsky performance status, serum lactate dehydrogenase measurement, hemoglobin measurement, and corrected serum calcium measurement). The multivariate analysis carried out on the K2 and TCGA series used the expression of the genes as a continuous variable with the log values of intensity and RPKM for the two datasets, respectively. Higher expression of these 8 genes resulted in a significantly decreased risk of death (HR 0.16 to 0.41 for K2 series and HR 0.68 to 0.84 for TCGA series). These included cysteine/tyrosine-rich 1 (*CYYR1*) and LIM domain binding 2 (*LDB2*) genes that have been recently reported as associated with disease-free survival with higher expression in tumours that metastasized after 24 months vs. tumours that metastasized earlier [30]. Furthermore, expression of sphingosine-1-phosphate receptor 1 (*S1PR1*), ephrin-B2 (*EFNB2*), G protein-coupled receptor 116 (*GPR116*), SNF related kinase (*SNRK*), transmembrane protein 204 (*TMEM204*), C-type lectin domain family 1, member A (*CLEC1A*) and C-type lectin domain family 14, member A (*CLEC14A*) was associated with increased survival time (Table 4). Finally, taking into consideration high correlation of genes (correlation >0.7 for K2 and TCGA datasets) the final list of genes was included together with the other covariates (age, tumour grade, pT and sex) in a multivariate Cox model and tested separately on each of the two datasets, with backwards step-wise selection to remove redundant genes. In each step, gene with the highest p-value was removed (significance level for removal from the model >0.2) resulting in a model that included *S1PR1* (for K2) and *S1PR1* and *CLEC1A* (for TCGA).

**Table 4:** Association of genes with overall survival in K2 and TCGA series.

	K2 Series–Czech Republic						TCGA Series–US					
	Univariate Analysis			Multivariate Analysis*			Univariate Analysis			Multivariate Analysis*		
Gene	HR	95% CI	P value	HR	95% CI	P value	HR	95% CI	P value	HR	95% CI	P value
**CLEC14A**	0.26	0.14–0.52	<0.001	0.41	0.19–0.9	0.025	0.72	0.64–0.81	<0.001	0.81	0.71–0.93	0.002
**CLEC1A**	0.08	0.02–0.29	<0.001	0.19	0.04–0.9	0.037	0.72	0.61–0.84	<0.001	0.83	0.69–1	0.049
**CYYR1**	0.18	0.08–0.39	<0.001	0.29	0.1–0.79	0.015	0.68	0.6–0.77	<0.001	0.79	0.69–0.91	0.001
**EFNB2**	0.19	0.08–0.43	<0.001	0.34	0.13–0.92	0.034	0.70	0.61–0.8	<0.001	0.81	0.69–0.95	0.008
**GPR116**	0.17	0.07–0.39	<0.001	0.30	0.11–0.8	0.016	0.69	0.61–0.78	<0.001	0.81	0.7–0.93	0.004
**LDB2**	0.24	0.13–0.46	<0.001	0.36	0.16–0.8	0.013	0.71	0.63–0.8	<0.001	0.82	0.71–0.95	0.009
**S1PR1**	0.07	0.02–0.27	<0.001	0.16	0.04–0.69	0.014	0.68	0.61–0.77	<0.001	0.79	0.69–0.9	<0.001
**TMEM204**	0.17	0.07–0.38	<0.001	0.31	0.11–0.85	0.023	0.74	0.65–0.84	<0.001	0.84	0.72–0.98	0.025

Results from univariate and multivariate Cox’s proportional hazard model analysis of prognostic factors for overall survival in K2 (expression microarrays) and TCGA (RNA-Seq) populations for a selection of genes. Abbreviations used: HR – hazard ratio; CI – confidence interval.

* Adjusted for age (continuous), grade, pT, sex.

## Discussion

Several microarray studies have been performed to detect gene expression signatures in renal cancer that would provide diagnostic and prognostic information [6], [17], [18], [31], [32], [33], [34], [35], [36], [37], [38]. However, most of the reports concentrate their efforts on a small number of cases from the US, Japanese and Western European populations while the highest incidence and mortality rates are found in Central and Eastern Europe. In this study, we investigated the gene expression and methylation profiles between ccRCC tumours and adjacent non-tumour tissue in relation to pathogenesis and clinical outcome of ccRCC in Czech Republic (K2 study) and in the US (TCGA data). This work represents one of the largest studies of gene expression using pairs of normal and tumour tissue of the conventional ccRCC subtype and to our knowledge the first report on molecular characterization of ccRCC in the Czech Republic.

The unsupervised clustering analysis of 101 Czech patients did not identify any clear molecular subgroups within tumour samples indicating molecular homogeneity of ccRCC samples used in our study. In recent years there has been an important development in our knowledge of ccRCC biology mainly through emerging molecular biology, genomic and transcriptomic techniques. Mutations in the *VHL* gene, observed in up to 80% of cases [39], resulting in overexpression of hypoxia-inducible factors (HIF) have been shown to play a fundamental role in the development of ccRCC. Animal studies have shown that activation of the HIF pathway (particularly HIF-2α) mediates the phenotypes observed in the context of *VHL* knock-out [40]. The HIF pathway further activates a range of adaptive tumour response genes involved in cell death, proliferation, cell differentiation, metabolism and angiogenesis (VEGF). Our results were consistent with these findings, with upregulation of both HIF-1α as well as HIF-2α target genes being consistently observed, including the most significant genes such as NADH dehydrogenase subcomplex *NDUFA4L2* [41], carbonic anhydrase 9 (*CA9*) [42] and vascular endothelial growth factor A (*VEGFA*) indicating the activation of HIF pathway. Moreover, we found other genes known to be involved in vasculature development and angiogenesis to be upregulated in ccRCC, comprising neuropilin 1 (*NRP1*), apolipoprotein L domain containing 1 (*APOLD1*), endothelin 1 (*EDN1*), notch 4 (*NOTCH4*), transforming growth factor, alpha (*TGFA*) and angiopoietin-related proteins (*ANGPT2, ANGPTL4*). We also identified significant increases in target genes specific to HIF-1α such as glucose transporter *SLC2A1* (*GLUT1*), lactate dehydrogenase A (*LDHA*) and mitochondrial protein encoding gene *BNIP3* as well as HIF-2α targeted genes: transforming growth factor, alpha (*TGFA*) and cyclin D1 (*CCND1*). While both HIF-1α and HIF-2α are key players in ccRCC pathogenesis, these two HIF-α isoforms have been shown to have different (suppressive) properties in RCC cells [43], with enhanced expression of HIF-2α suppressing HIF-1α and vice-versa. In this context, two subtypes of ccRCC have been proposed: a subtype distinguished by overexpression of both HIF-1α and HIF-2α (H1H2) and another expressing HIF-2α (H2). These classifications demonstrate different gene expression patterns, varying clinical outcomes and possible distinct targeted therapies needed to treat these tumours [44]. Furthermore, both HIF-1α and its target -CA9 expression has been related to worse survival and reported as independent prognostic factors in metastatic ccRCC [42].

The HIF pathway also plays a role in the cellular response to stress, such as metabolic, hypoxic, or inflammatory stress. Inflammatory signalling and infiltration are key factors in tumour progression. HIF-1α and HIF-2α with their opposing and overlapping functions in tumour cells as well as in inflammatory cells of the tumour microenvironment can crosstalk between these populations and have clear effects on tumour metabolism, inflammation, and progression [45]. Recent studies have also shown a link between HIF signalling and pro-inflammatory transcription factor nuclear factor kappa B (NF-κB) during inflammation [46]. The NF-κB pathway plays a central role in the regulation of immune responses and targets several inflammatory cytokines, such as TNF-α, IL-1, IL-6 and IL-8 and its aberrant activation has been identified in many cancer types. In our study several immune response and inflammatory genes were found to be upregulated in ccRCC, including *IL2RA, IL2RB, IL7R, IL10RB* as well as tumour necrosis factor family genes *TNFAIP3, TNFAIP6, TNFAIP8, TNFRSF4*.

In addition, a panel of inflammation related genes has recently been analyzed in ccRCC by Tan and colleagues and related to risk of recurrence (*GADD45G*) and death (*CARD9, CIITA* and *NCF2*) [15]. Only one report highlights the role of Toll-like receptors (TLRs) in ccRCC. TLRs are molecules in the innate immune system for which targeted immunotherapy is under development. Overexpression of *TLR3* in both primary and metastatic ccRCC tissues has been described by Morikawa *et al.* [47] and has been shown to induce type I IFN production and NF-κB activation. Here we showed upregulation of both *TLR3* and *TLR7*. These indicate possible mutual interactions and regulation of TLRs-NF-κB – HIF – VEGF pathways and their downstream targets in ccRCC tumourigenesis. These pathways as well as innate and adaptive immune responses demonstrated by T-cell activation have become an area of active research, with antibodies against CTLA-4 and PD-1 showing clinical efficacy and anti-tumour activity. New therapies targeting molecular pathways involved in ccRCC pathogenesis, such as agents targeting downstream genes of the VHL/HIF pathway -tyrosine kinase and mTOR inhibitors, have also shown encouraging effects. To complement search for potential therapeutic targets, we performed Connectivity Map analysis on the most differentially expressed genes (http://www.broadinstitute.org/cmap/). Using this approach we identified additional compounds with a potential for ccRCC targeted therapy including carbonic anhydrase and topoisomerase inhibitors (data not shown).

Based on the similarity in gene expression profiles between the Czech and US population we conclude that the molecular features of ccRCC in both locations are largely overlapping and share the same molecular abnormalities. We also observed, as previously reported [48], that the RNA-Seq platform was more sensitive in identifying significantly differentially expressed genes than the microarray platform as reflected in superior fold changes. However, we found an overlap of more than 60% of differentially expressed genes in two different populations whilst applying two different high-throughput technologies.

Latest reports on recurrent mutations in genes encoding several epigenetic regulators such as SWI/SNF complex gene *PBRM1* [8], methyltransferases *SETD2* and *MLL2*, demethylases *UTX* (*KDM6A*) and *JARID1C* (*KDM5C*) [6] suggest possible implications of epigenetic variation in ccRCC. The methylation status of individual genes has been examined in normal and renal tumour cells and, more recently, global profiles of gene methylation of RCC have been studied in cell lines [49]. We analyzed 317 ccRCC and adjacent non-tumour renal tissues from genome-wide DNA methylation data from TCGA and compared it to our gene expression study. Our results confirmed hypomethylation of *CA9* [50] and identified new, at the time of preparation of the manuscript, hypomethylated genes known to be overexpressed in ccRCC, including nicotinamide N-methyltransferase (*NNMT*), caveolin 1 (*CAV1*) and cyclin D1 (*CCND1*). Thereafter, Girgis and colleagues also reported on hypomethylation of *NNMT* and *CCND1* genes in ccRCC [51]. Furthermore, the increased mRNA levels of immune response genes (*ENPP3, LCP1, HLA-DPA1, HLA-DRA, HLA-DMA, TNFSF13B, GBP4, PAG1, SLA, AQP9, CD37, IL7R*), genes involved in signal transduction (*RGS1, KSR1, ITGAX, CD53, IFNGR2, S100A10*), cell proliferation *(CCND1, ISG20, SPARC, RUNX3*) and cell death (*LGALS1, PLEK, IFI16, NOL3, CD14, ADA, HSPB8, GZMH*) could be partially attributed to changes in methylation status. Downregulated genes associated with ccRCC *AQP2* and *SERPINA5* were found to be hypermethylated. Other genes of which expression could be modulated by hypermethylation events included cellular transport genes (*AQP2, KCNJ1, CLCNKB, SCNN1A, SLC12A3, ATP1A1, CEL, RAB25*), homeostasis (*PTH1R*, *ATP6V0A4*, *CLDN16*), cellular responses (*ALDOB*, *MUC1*, *RBP4*), adhesion (*CLDN8*, *CYFIP2*, *FBLN5*, *CLDN19*), development (*SFRP1*, *PLXNB1*, *SEMA3B*, *ANK3*) and cellular and metabolic processes (*SERPINA5*, *MT1E*, *TACSTD2*, *TSPAN8*, *GGT6*, *HRG*, *CEL*, *CKMT2*). To our knowledge this is the first report on methylation status of these genes in ccRCC.

Another novel finding of this study is the identification of gene expression levels independently associated with the overall survival. Multivariate Cox proportional-hazard analysis with adjustment for age, tumour grade, extension of primary tumour and sex was employed to correlate gene expression with survival time. Our study identified a signature of eight genes with highly correlated expression that was associated with ccRCC prognosis. Expression of each of the genes in this signature was associated with prolonged survival in the K2 and TCGA sets. The signature included *CYYR1* and *LDB2* genes that have been described to be correlated with disease-free survival in synchronously vs. metachronously metastasized primary ccRCC, and in synchronous vs. metachronous metastases [30]. Downregulation of *S1PR1* expression has been shown to increase proliferative activity resulting in enhanced malignancy and poor survival of glioblastomas [52] while high-level expression of transcripts encoding ephrin-B2 has been reported as predictive of favourable disease outcome of neuroblastoma [53]. The angiogenesis-related gene *CLEC14A (EGFR5)* and immune response gene *CLEC1A* were found in our study to be associated with longer survival. *CLEC14A* (*EGFR5*) plays a role in cell-cell adhesion and angiogenesis, because of its presence at higher levels in tumour endothelium it has been considered to be a candidate for tumour vascular targeting [54]. Furthermore, *GPR116* gene associated with overall survival in our study have been already described to be overexpressed in lung adenocarcinoma [55]. Finally, we identified *TMEM204* (*CLP24*), a hypoxically regulated intercellular junction protein [56], as predictive of overall survival. In summary, if validated in independent series and by further protein assays, some or all of the 9 genes may represent potential candidate biomarkers that can predict independently of grade the outcome in patients with ccRCC. These results indicate that an algorithm based on expression data from a subgroup of genes may form the basis of a potential prognostic tool. Further definition and validation of such an algorithm is required and other genes significant in the TCGA dataset but not in the Czech data (Table S8, File S2) including cyclins (A2, B2), *BUB1, BIRC5, AURKB, EPAS1* among others should not be discarded if validated in larger cohorts.

In summary, we confirmed alterations in HIF pathway (through upregulation of HIF-1α and HIF-2α target genes) and in the signalling pathways upstream and downstream of HIF in the Czech Republic population. We also reported on the methylation status of genes involved in ccRCC pathogenesis and identified new genes potentially associated with prolonged survival.

## Materials and Methods

### Patient Population and Biosample Collection and Processing

#### Patient recruitment and biosample collection

As part of the ongoing case-control “K2 study”, renal cancer patients have been recruited in four areas of Czech Republic (Prague, Olomouc, Ceske Budejovice and Brno) between 2008 and 2012. Interviewers were trained in each centre to perform face-to-face interviews with cases (at the hospital) using standard questionnaires that covered tobacco use, alcohol consumption, body mass, medical history, and family history of cancer. Clinical and pathological information was abstracted from medical records, including clinical and pathological stages, tumour size, grade, histological type, treatment, tumour progression, relapse, and survival.

Tumour and adjacent non-tumour tissue samples were obtained from newly diagnosed patients who underwent partial or radical nephrectomy for a clinically diagnosed renal cancer. All samples were preserved in RNA*later*® solution and stored frozen at −20°C until tissue sectioning and RNA extraction. For this study, we selected 148 patients with histologically confirmed diagnosis of ccRCC, and with a complete set of samples, demographic and lifestyle data.

#### Ethics statement

The study protocol was approved by the institutional review boards of the International Agency for Research on Cancer and all collaborating centres/institutions (Regional Hospital (Ceske Budejovice), General Teaching Hospital and University Hospital Motol (Prague), Masaryk Memorial Cancer Institute (Brno), and Palacky University (Olomouc)) and written informed consent was obtained for all participating subjects.

#### Biosample processing and pathological examination

Tissue samples were embedded in Optimal Cutting Temperature (OCT) compound and processed to obtain consecutively one 5 µm section placed on slide and stained with haematoxylin and eosin (H&E), two 20 µm sections for RNA extraction, two 20 µm sections as a backup, and another 5 µm H&E stained section. For each tumour tissue, H&E sections were examined by a pathologist (BAA) independent from the pathologist who established the initial diagnosis to (i) confirm the ccRCC tumour type, and (ii) assess the tumour cell contents among viable cells present in the tissue. Non-tumour tissue H&E sections were also examined to confirm the non-tumour nature of collected paired samples. This thorough pathological examination led to the exclusion of 9 cases due to low tumour cell contents (<30%), leaving 139 (94%) cases for the purpose of the study.

#### RNA extraction

Total RNA was extracted from fresh frozen tumour and normal samples using Total RNA Isolation NucleoSpin® RNA II kit (Macherey Nagel) according to manufacturer’s instructions. RNA integrity (RIN) was assessed on an Agilent 2100 Bioanalyzer using the Agilent RNA 6000 Nano Kit (Agilent Technologies, Santa Clara, CA). Out of 139 sample pairs, we excluded 26 (19%) pairs with RIN value<6 for RNA extracted from the tumour sample (N = 4), the non-tumour sample (N = 21), or both (N = 1), leaving 103 cases for the analysis. On average, RIN values for the included samples were 8.4 for tumour samples and 7.3 for non-tumour samples.

### Microarray Hybridization and Data Analysis

500ng of mRNA was amplified into cRNA and biotinylated cRNA using Illumina® TotalPrep™-24 RNA Amplification Kit (Life Technologies) for the first batch, and two Illumina® TotalPrep™-96 RNA Amplification for the second and third batches (batches 2 and 3 included 2 technical replicate pairs from batch 1, and batch 3 included an additional technical replicate from batch 2). Subsequent steps included hybridization of each sample to Illumina HumanHT-12 v4 Expression BeadChips, washing, blocking, and streptavidin-Cy3 staining.

Illumina’s GenomeStudio software was used to generate signal intensity values from the scans and perform the initial quality controls. We found high correlation coefficients within the 5 technical replicate pairs (Pearson correlation range: 0.96–0.98), indicating a good repeatability of our experiments. The performance of hybridizations was evaluated by assessing the presence of outliers (very low or very high average signal intensity) and the noise-to-signal ratios by calculating P95/P05 ratios for each sample. An outlier was defined as a sample with P95/P05 ratio <9.5 and array intensity distribution distant from the rest of the data as identified by MDS plots (PC >50 and PC<−50) and density plots following samples normalization. One tumour and one non-tumour samples were found to be outliers and the corresponding pairs were excluded from the analysis. All samples were found to show an acceptable noise-to-signal ratio (P95/P05>9.6). Supplemental quality assessment (before and after normalization) was conducted using arrayQualityMetrics package [57] and reached similar conclusions. In total, 101 sample pairs were then included in the analysis.

Table 1 summarizes the clinical and demographic characteristics of this K2 series of 101 patients, which included 59 men and 42 women. The majority of cases were between 55 and 74 years of age and no significant differences were observed between male and female individuals for known ccRCC risk factors.

All statistical analyses were performed using R Bioconductor software, with the addition of the BRBArray tool for specific applications. The raw signal intensities of all samples were processed with lumi package applying variance-stabilizing transformation (VST) and quantile normalization [58]. Linear Models for Microarray Analysis (limma package) using the paired t-test method was used for identification of differentially expressed probes [59]. Significance levels (*P* values) were corrected using Benjamini and Hochberg (BH) false discovery rate (FDR) method to correct for multiple hypothesis testing. Probes with a FDR-adjusted p-value of <0.05 and probes with a minimum of 2-fold change were considered significantly differentially expressed. Unsupervised Hierarchical Clustering was performed with the average linkage method while the differential gene expression was measured using centered correlation technique implemented in BRBArray tool. The Database for Annotation, Visualization and Integrated Discovery (DAVID) v 6.7 was used for classification of the differentially expressed genes according to biological and molecular processes [60]. Gene Set Enrichment Analysis (GSEA) (Broad Institute) was used to evaluate the combined significance of sets of genes in ccRCC using annotations from a curated version of Biocarta. Only gene sets represented by at least 15 genes were retained. Genes were ranked based on the limma-moderated *t* statistic. After Kolmogorov–Smirnoff testing, those gene sets showing FDR <0.25, a well-established cut-off for the identification of biologically relevant gene sets [22] were considered enriched between classes under comparison. The microarray experiments are MIAME compliant and have been deposited at the NCBI Gene Expression Omnibus (GEO) database (http://www.ncbi.nlm.nih.gov/geo) under accession GSE40435.

### Secondary Analysis of Data from the Cancer Genome Atlas (TCGA)

TCGA RNA-Seq (Level 3), methylation (Level 1) and corresponding clinical data were downloaded from TCGA website https://tcga-data.nci.nih.gov/tcga/following approval of this project by the consortium. RNA-Seq analysis used data from 65 tumour/non-tumour tissue pairs and 400 unpaired tumour tissue samples. Methylation analysis were based on data from 188 tumour/non-tumour tissue pairs analyzed on the Illumina Infinium HumanMethylation 27K BeadChip assay and 129 additional pairs analyzed on the Infinium HumanMethylation 450K BeadChip assay. A description of the TCGA cases used in this study is available in File S1.

#### RNA-Seq data analysis

For RNA-Seq analysis, TMM normalization [24] available in EdgeR package was applied to RPKM values and the voom function was used to convert the values to log2-cpm, with associated weights. Differential expression analysis between tumour and non-tumour tissue (paired t-test) was performed with the limma package implemented in Bioconductor [61] using uniquely mapped RPKM per gene as input. Only transcripts that were found to be differentially expressed with an FDR-adjusted p-value <0.05 (adjusted using a BH method for multiple testing [62]) and with a minimum of 2-fold change were considered significant and used for downstream analysis. Up -and down -regulated genes were considered independently and comparison with microarray-based results from the Czech data was illustrated with Venn diagrams (http://bioinfogp.cnb.csic.es/tools/venny/index.html).

#### Methylation data analysis

Raw data were imported with methylumi package and Bioconductor lumi package was used to process both Illumina Infinium HumanMethylation 27K and 450K DNA methylation data. The data was first subjected to a QA/QC step (boxplot, density plot of M-values, principal component analysis). Following removal of outliers (defined by array intensity distribution distant from the rest of the data as identified by MDS plots (PC >50 and PC<−50) and density plots following samples normalization), we performed a colour balance adjustment of methylated and unmethylated probe intensities between the two colour channels using a smooth quantile normalization method. The methylation M-value (log2 ratio of methylated and unmethylated probes) was calculated to estimate the methylation level of the measured CpG sites [63]. The follow-up analysis was then based on the M-value. We used a stringent quantile normalization method. The assumption of “quantile” normalization is that the intensity distribution of the pooled methylated and unmethylated probes is similar for different samples. Following this pre-processing, the differential analysis of methylation data was similar to that used for expression microarray data. To compare the differences in methylation between tumour and adjacent non-tumour tissues, we performed differential analyses using routines implemented in the limma package (paired t-test). To ensure both statistical significance and strong biological effects, we required that the differentially methylated CpG sites had an FDR <0.05 and a minimum of 2-fold change (based on M-value). Using this approach, 1811 CpG sites (916 up, 895 down) and 35552 CpG sites (16469 up, 19083 down) were identified for Illumina Infinium HumanMethylation 27 k and 450 k, respectively. The differentially methylated CpG sites common to both platforms were mapped to differentially expressed genes.

### Survival Analysis

To identify sets of genes related to ccRCC prognosis, the “Filter, cluster, and stepwise model selection” (FCMS) method [64] implemented in the web tool SignS (http://signs.bioinfo.cnio.es) [27] was used. Briefly, individual genes were tested for association with prognosis using a univariate Cox regression. Only genes with a nominal p-value <0.001 were retained for further analyses. Genes were then divided into two groups, those with a positive and negative coefficient in the Cox model and clustered separately. A cluster was restricted to contain between 10 and 100 genes with minimum correlation coefficient of 0.8. All possible pairs of signatures were then jointly fitted with a Cox model. Stepwise variable selection using the best two-signature model was performed using the AIC criterion. Final assessment of the significance of association was performed by splitting the samples into several (2, 3 or 4) groups based on their predicted scores, and comparing survival functions of these groups (better vs. worse prognosis, tertiles, quartiles). The predicted scores were obtained from a 10-fold cross validation. Since the gene expression microarray K2 series was smaller (89 ccRCC cases), had better survival (12 deaths) and shorter follow-up time, reducing the power of tests based on this data alone, the performance of the final model was evaluated against the larger TCGA series (464 ccRCC cases). In this analysis the TCGA series was used to assess predictive performance, and not to build the model. In addition, to evaluate whether important features were missed in the smaller gene expression microarray K2 series, we build a separate model using only the TCGA series, with significance assessed by cross-validation, and examined the overlap of the sets of selected genes. Finally, the genes selected in the FCMS method were individually tested in a further Cox model (using STATA v 11 statistical software), where additional covariates (age, sex, tumour grade and extent of primary tumour) were included, to guard against possible confounding. Those genes not significantly associated (p>0.05) in this test were discarded from the model. Finally, the list of significant genes was then included together with the other covariates in a final multivariate Cox model and tested separately on each of the two datasets, with backwards step-wise selection used to remove redundant genes.

## Supporting Information

Figure S1 **Unsupervised Hierarchical Clustering of tumour and non-tumour samples separately following quantile normalization showing no identifiable cluster.**
(TIF)

Figure S2 **Detailed workflow for RNA-Sequencing data analysis including data processing prior to download (A) and data normalization and transformation (B).**
(TIF)

Table S1 **Complete list of statistically significant differentially expressed probes (FDR-adjusted p-value (BH) <0.05, FC ≥2) in ccRCC as compared with adjacent non-tumour tissues in Czech Republic (K2 series)–whole-genome expression profiling using microarrays.**
(XLS)

Table S2 **Enriched GO categories among the transcripts significantly differentially expressed between ccRCC tumour and adjacent non-tumour tissues in microarray analysis of K2 series using DAVID tools.**
(XLS)

Table S3 **List of pathways enriched in ccRCC phenotype identified in gene set enrichment analysis (GSEA).** All gene sets are derived from Biocarta pathway database.
(XLSX)

Table S4 **Complete list of statistically significant differentially expressed genes (FDR-adjusted p-value (BH) <0.05, FC ≥2) in ccRCC as compared with adjacent non-tumour tissues in US population (TCGA series) – RNA-Sequencing analysis.**
(XLS)

Table S5 **Enriched GO categories among the transcripts significantly differentially expressed between ccRCC tumour and adjacent non-tumour tissues in RNA-Sequencing analysis of TCGA series using DAVID tools.**
(XLS)

Table S6 **Complete list of statistically significant differentially hypermethylated and hypomethylated CpG sites (FDR-adjusted p-value (BH) <0.05, FC ≥2) in ccRCC as compared with adjacent non-tumour tissues in US population (TCGA series) using Illumina Infinium HumanMethylation 27K and 450K BeadChip assays.**
(XLSX)

Table S7 **Complete list of statistically significant differentially expressed probes (FDR-adjusted p-value (BH) <0.05, FC ≥2) in grades 1–4 separately for ccRCC as compared with adjacent non-tumour tissues in Czech Republic (K2 series) – whole-genome expression profiling using microarrays.**
(XLSX)

Table S8 **Results from univariate and multivariate Cox’s proportional hazard model analysis of all prognostic factors for overall survival in K2 (expression microarrays) and TCGA (RNA-Seq) populations identified in SignS analysis.**
(DOCX)

File S1 **List of the Cancer Genome Atlas (TCGA) project cases used for the analysis.**
(XLS)

File S2 **List of genes identified as associated with overall survival in both K2 (Czech Republic) and TCGA (US) datasets or TCGA dataset alone using SignS software.**
(XLS)
