# Supplementary figures and images for: Integrative Genome-Wide Gene Expression Profiling of Clear Cell Renal Cell Carcinoma in Czech Republic and in the United States
Source: PLoS One. 2013 Mar 5;8(3):e57886. doi: 10.1371/journal.pone.0057886 (PMC3589490; doi:10.1371/journal.pone.0057886)

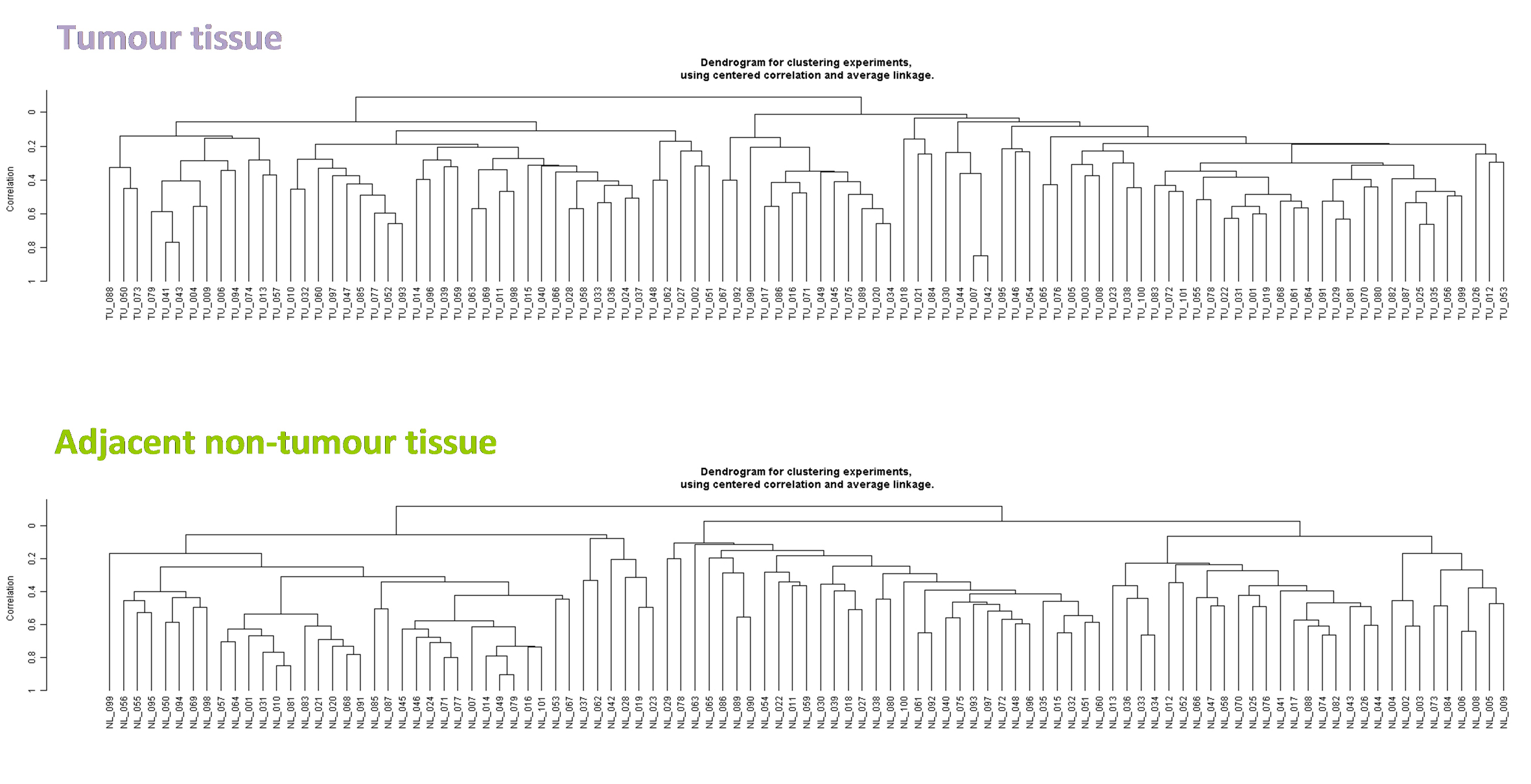

Supplement: Figure S1 — Unsupervised Hierarchical Clustering of tumour and non-tumour samples separately following quantile normalization showing no identifiable cluster. (TIF) [file pone.0057886.s001.tif]

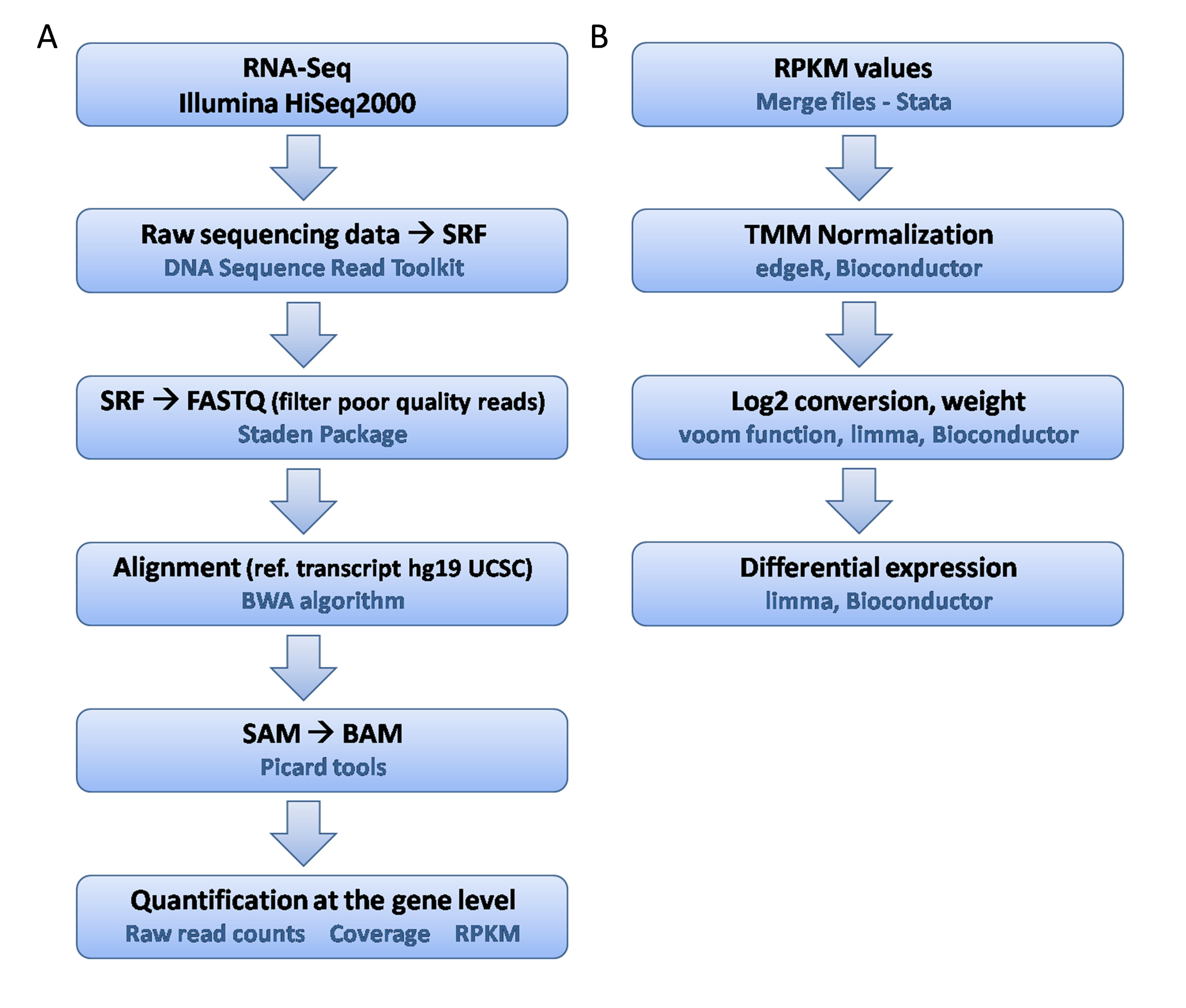

Supplement: Figure S2 — Detailed workflow for RNA-Sequencing data analysis including data processing prior to download (A) and data normalization and transformation (B). (TIF) [file pone.0057886.s002.tif]
