# Supplementary material for: Integrative Genome-Wide Gene Expression Profiling of Clear Cell Renal Cell Carcinoma in Czech Republic and in the United States
Source: PLoS One. 2013 Mar 5;8(3):e57886. doi: 10.1371/journal.pone.0057886 (PMC3589490; doi:10.1371/journal.pone.0057886)
Supplement: Table S8 — Results from univariate and multivariate Cox’s proportional hazard model analysis of all prognostic factors for overall survival in K2 (expression microarrays) and TCGA (RNA-Seq) populations identified in SignS analysis. (DOCX) [file pone.0057886.s010.docx]

|  | **K2 Series - Czech Republic** | | | | | | **TCGA Series - US** | | | | | |
| --- | --- | --- | --- | --- | --- | --- | --- | --- | --- | --- | --- | --- |
|  | **Univariate Analysis** | | | **Multivariate Analysis*** | | | **Univariate Analysis** | | | **Multivariate Analysis*** | | |
| **Gene** | **HR** | **95% CI** | **P value** | **HR** | **95% CI** | **P value** | **HR** | **95% CI** | **P value** | **HR** | **95% CI** | **P value** |
| ADCY4 | 0.19 | 0.07 - 0.49 | 0.001 | 0.28 | 0.09 - 0.89 | 0.031 | 0.89 | 0.76 - 1.03 | 0.120 | 1.02 | 0.86 - 1.22 | 0.815 |
| ARAP3 | 0.15 | 0.05 - 0.45 | 0.001 | 0.26 | 0.06 - 1.07 | 0.063 | 0.88 | 0.74 - 1.05 | 0.164 | 1.08 | 0.88 - 1.34 | 0.456 |
| ASPM | 3.47 | 1.63 - 7.35 | 0.001 | 1.80 | 0.52 - 6.27 | 0.354 | 1.35 | 1.17 - 1.55 | < 0.001 | 1.19 | 1.03 - 1.37 | 0.016 |
| AURKB | 7.51 | 2.2 - 25.66 | 0.001 | 1.50 | 0.23 - 9.63 | 0.670 | 1.55 | 1.37 - 1.76 | < 0.001 | 1.30 | 1.13 - 1.5 | < 0.001 |
| BIRC5 | 14.98 | 3.64 - 61.69 | < 0.001 | 2.31 | 0.21 - 25.08 | 0.493 | 1.60 | 1.39 - 1.84 | < 0.001 | 1.33 | 1.14 - 1.55 | < 0.001 |
| CCNA2 | 8.28 | 2.42 - 28.31 | 0.001 | 1.39 | 0.17 - 11.39 | 0.757 | 1.67 | 1.4 - 1.99 | < 0.001 | 1.33 | 1.1 - 1.6 | 0.003 |
| CCNB2 | 3.94 | 1.98 - 7.82 | < 0.001 | 2.05 | 0.55 - 7.67 | 0.288 | 1.60 | 1.38 - 1.86 | < 0.001 | 1.31 | 1.11 - 1.54 | 0.001 |
| CD34 | 0.06 | 0.01 - 0.26 | < 0.001 | 0.18 | 0.02 - 1.33 | 0.093 | 0.71 | 0.62 - 0.81 | < 0.001 | 0.79 | 0.68 - 0.92 | 0.002 |
| CDC20 | 3.49 | 1.84 - 6.62 | < 0.001 | 2.16 | 0.69 - 6.71 | 0.184 | 1.58 | 1.38 - 1.81 | < 0.001 | 1.31 | 1.13 - 1.52 | < 0.001 |
| CDCA5 | 7.09 | 2.72 - 18.5 | < 0.001 | 2.44 | 0.48 - 12.47 | 0.286 | 1.45 | 1.23 - 1.71 | < 0.001 | 1.21 | 1.01 - 1.44 | 0.034 |
| CDCA8 | 10.33 | 2.71 - 39.37 | 0.001 | 1.00 | 0.08 - 12.15 | 0.998 | 1.59 | 1.35 - 1.86 | < 0.001 | 1.27 | 1.06 - 1.52 | 0.008 |
| CDH5 | 0.26 | 0.13 - 0.54 | < 0.001 | 0.48 | 0.21 - 1.11 | 0.087 | 0.72 | 0.63 - 0.83 | < 0.001 | 0.81 | 0.7 - 0.95 | 0.009 |
| CENPF | 5.76 | 2.42 - 13.71 | < 0.001 | 3.65 | 0.58 - 22.85 | 0.167 | 1.49 | 1.28 - 1.75 | < 0.001 | 1.25 | 1.06 - 1.47 | 0.007 |
| CEP55 | 4.05 | 2.07 - 7.94 | < 0.001 | 2.49 | 0.67 - 9.29 | 0.175 | 1.52 | 1.31 - 1.75 | < 0.001 | 1.26 | 1.07 - 1.48 | 0.006 |
| CKAP2L | 11.25 | 3.38 - 37.4 | < 0.001 | 5.25 | 0.58 - 47.27 | 0.139 | 1.26 | 1.08 - 1.46 | 0.003 | 1.12 | 0.97 - 1.3 | 0.126 |
| CLEC14A | 0.26 | 0.14 - 0.52 | < 0.001 | 0.41 | 0.19 - 0.9 | 0.025 | 0.72 | 0.64 - 0.81 | < 0.001 | 0.81 | 0.71 - 0.93 | 0.002 |
| CLEC1A | 0.08 | 0.02 - 0.29 | < 0.001 | 0.19 | 0.04 - 0.9 | 0.037 | 0.72 | 0.61 - 0.84 | < 0.001 | 0.83 | 0.69 - 1 | 0.049 |
| CYYR1 | 0.18 | 0.08 - 0.39 | < 0.001 | 0.29 | 0.1 - 0.79 | 0.015 | 0.68 | 0.6 - 0.77 | < 0.001 | 0.79 | 0.69 - 0.91 | 0.001 |
| DLGAP5 | 8.50 | 2.32 - 31.19 | 0.001 | 0.91 | 0.1 - 8.59 | 0.932 | 1.40 | 1.21 - 1.63 | < 0.001 | 1.19 | 1.02 - 1.39 | 0.023 |
| EFNB2 | 0.19 | 0.08 - 0.43 | < 0.001 | 0.34 | 0.13 - 0.92 | 0.034 | 0.70 | 0.61 - 0.8 | < 0.001 | 0.81 | 0.69 - 0.95 | 0.008 |
| ESAM | 0.30 | 0.15 - 0.58 | < 0.001 | 0.54 | 0.24 - 1.22 | 0.141 | 0.73 | 0.63 - 0.84 | < 0.001 | 0.84 | 0.71 - 0.99 | 0.036 |
| GPR116 | 0.17 | 0.07 - 0.39 | < 0.001 | 0.30 | 0.11 - 0.8 | 0.016 | 0.69 | 0.61 - 0.78 | < 0.001 | 0.81 | 0.7 - 0.93 | 0.004 |
| GRRP1 | 0.04 | 0.01 - 0.23 | < 0.001 | 0.12 | 0.02 - 0.78 | 0.026 | 0.78 | 0.67 - 0.9 | 0.001 | 0.89 | 0.76 - 1.05 | 0.167 |
| HJURP | 6.88 | 2.29 - 20.67 | 0.001 | 1.10 | 0.17 - 6.9 | 0.920 | 1.89 | 1.62 - 2.22 | < 0.001 | 1.57 | 1.31 - 1.87 | < 0.001 |
| HMMR | 5.08 | 1.89 - 13.63 | 0.001 | 1.36 | 0.26 - 7.09 | 0.712 | 1.33 | 1.14 - 1.54 | < 0.001 | 1.15 | 0.99 - 1.35 | 0.073 |
| JAM2 | 0.06 | 0.01 - 0.3 | 0.001 | 0.19 | 0.03 - 1.02 | 0.053 | 0.76 | 0.65 - 0.88 | < 0.001 | 0.88 | 0.75 - 1.03 | 0.113 |
| JAM3 | 0.24 | 0.11 - 0.55 | 0.001 | 0.44 | 0.16 - 1.25 | 0.124 | 0.74 | 0.63 - 0.87 | < 0.001 | 0.85 | 0.71 - 1.02 | 0.080 |
| KIF11 | 6.82 | 2.32 - 20.07 | < 0.001 | 1.80 | 0.21 - 15.5 | 0.592 | 1.57 | 1.28 - 1.93 | < 0.001 | 1.29 | 1.04 - 1.6 | 0.023 |
| KIF14 | 19.19 | 3.59 - 102.46 | 0.001 | 4.58 | 0.37 - 57.08 | 0.237 | 1.58 | 1.34 - 1.86 | < 0.001 | 1.35 | 1.14 - 1.6 | 0.001 |
| KIF20A | 4.57 | 2 - 10.42 | < 0.001 | 2.14 | 0.49 - 9.33 | 0.311 | 1.50 | 1.32 - 1.72 | < 0.001 | 1.25 | 1.08 - 1.45 | 0.002 |
| KIF23 | 157.39 | 11.84 - 2092.78 | < 0.001 | 13.14 | 0.07 - 2542.47 | 0.338 | 1.71 | 1.45 - 2.01 | < 0.001 | 1.40 | 1.17 - 1.68 | < 0.001 |
| KIF2C | 13.57 | 3.2 - 57.63 | < 0.001 | 3.20 | 0.18 - 56.52 | 0.427 | 1.62 | 1.4 - 1.88 | < 0.001 | 1.33 | 1.13 - 1.56 | 0.001 |
| KIFC1 | 4.16 | 1.86 - 9.29 | 0.001 | 1.84 | 0.34 - 9.98 | 0.482 | 1.57 | 1.32 - 1.88 | < 0.001 | 1.29 | 1.07 - 1.56 | 0.007 |
| LDB2 | 0.24 | 0.13 - 0.46 | < 0.001 | 0.36 | 0.16 - 0.8 | 0.013 | 0.71 | 0.63 - 0.8 | < 0.001 | 0.82 | 0.71 - 0.95 | 0.009 |
| MELK | 4.95 | 1.96 - 12.47 | 0.001 | 1.60 | 0.32 - 8.15 | 0.569 | 1.74 | 1.44 - 2.1 | < 0.001 | 1.42 | 1.16 - 1.74 | 0.001 |
| NCAPG | 5.29 | 2.03 - 13.77 | 0.001 | 1.18 | 0.21 - 6.49 | 0.849 | 1.68 | 1.39 - 2.03 | < 0.001 | 1.31 | 1.08 - 1.61 | 0.008 |
| PRC1 | 5.60 | 2.44 - 12.85 | < 0.001 | 3.27 | 0.61 - 17.61 | 0.167 | 1.66 | 1.33 - 2.06 | < 0.001 | 1.42 | 1.14 - 1.76 | 0.002 |
| PTTG1 | 3.10 | 1.77 - 5.43 | < 0.001 | 2.39 | 0.85 - 6.71 | 0.098 | 1.58 | 1.37 - 1.82 | < 0.001 | 1.28 | 1.08 - 1.5 | 0.003 |
| PTTG3P | 2.75 | 1.62 - 4.66 | < 0.001 | 2.02 | 0.77 - 5.29 | 0.151 | 1.10 | 0.92 - 1.31 | 0.299 | 1.03 | 0.86 - 1.23 | 0.745 |
| RASIP1 | 0.14 | 0.06 - 0.33 | < 0.001 | 0.26 | 0.09 - 0.75 | 0.013 | 0.72 | 0.62 - 0.84 | < 0.001 | 0.85 | 0.72 - 1.01 | 0.073 |
| S1PR1 | 0.07 | 0.02 - 0.27 | < 0.001 | 0.16 | 0.04 - 0.69 | 0.014 | 0.68 | 0.61 - 0.77 | < 0.001 | 0.79 | 0.69 - 0.9 | < 0.001 |
| SH2D3C | 0.01 | 0 - 0.09 | < 0.001 | 0.04 | 0 - 0.55 | 0.017 | 0.84 | 0.71 - 1 | 0.047 | 0.91 | 0.74 - 1.12 | 0.372 |
| SNRK | 0.06 | 0.02 - 0.22 | < 0.001 | 0.11 | 0.02 - 0.55 | 0.007 | 0.65 | 0.54 - 0.79 | < 0.001 | 0.83 | 0.67 - 1.03 | 0.094 |
| SOX17 | 0.02 | 0 - 0.16 | < 0.001 | 0.05 | 0 - 0.66 | 0.023 | 0.74 | 0.64 - 0.87 | < 0.001 | 0.87 | 0.73 - 1.03 | 0.098 |
| SPC24 | 5.17 | 2.24 - 11.96 | < 0.001 | 1.41 | 0.33 - 5.96 | 0.639 | 1.53 | 1.3 - 1.8 | < 0.001 | 1.31 | 1.11 - 1.54 | 0.001 |
| TMEM204 | 0.17 | 0.07 - 0.38 | < 0.001 | 0.31 | 0.11 - 0.85 | 0.023 | 0.74 | 0.65 - 0.84 | < 0.001 | 0.84 | 0.72 - 0.98 | 0.025 |
| TOP2A | 3.31 | 1.64 - 6.69 | 0.001 | 1.51 | 0.48 - 4.73 | 0.478 | 1.39 | 1.18 - 1.63 | < 0.001 | 1.19 | 1.01 - 1.39 | 0.036 |
| TPX2 | 6.02 | 2.28 - 15.88 | < 0.001 | 3.03 | 0.57 - 16.14 | 0.193 | 1.55 | 1.35 - 1.79 | < 0.001 | 1.29 | 1.1 - 1.51 | 0.002 |
| TTK | 10.31 | 2.96 - 35.96 | < 0.001 | 4.85 | 0.5 - 47.31 | 0.175 | 1.52 | 1.29 - 1.8 | < 0.001 | 1.27 | 1.06 - 1.51 | 0.009 |
| UBE2C | 7.30 | 2.63 - 20.23 | < 0.001 | 2.11 | 0.25 - 18.06 | 0.496 | 1.47 | 1.33 - 1.64 | < 0.001 | 1.24 | 1.1 - 1.39 | 0.001 |
| UBE2T | 5.08 | 2.04 - 12.68 | < 0.001 | 1.82 | 0.41 - 8.07 | 0.430 | 1.81 | 1.5 - 2.19 | < 0.001 | 1.40 | 1.13 - 1.72 | 0.002 |

* Adjusted by age (continuous), grade, pT, sex
